# Supplementary material for: Antimicrobial Activity of Selenium Nanoparticles (SeNPs) against Potentially Pathogenic Oral Microorganisms: A Scoping Review
Source: Pharmaceutics. 2023 Aug 31;15(9):2253. doi: 10.3390/pharmaceutics15092253 (PMC10537110; doi:10.3390/pharmaceutics15092253)
Supplement: Supplementary file 1 [file pharmaceutics-15-02253-s001.zip › pharmaceutics-2501888-supplementary.pdf]

Antimicrobial activity of Selenium nanoparticles (SeNPs) against potentially pathogenic oral microorganisms: A Scoping Review

Eulàlia Sans-Serramitjana[1*], Macarena Obreque[2], Fernanda Muñoz[2], Carlos Zaror[3,4], María de La Luz Mora[1], Miguel Viñas[5], Pablo Betancourt[2,6**]

[1]Center of Plant, Soil Interaction and Natural Resources Biotechnology, Scientific and Biotechnological Bioresource Nucleus (BIOREN-UFRO), Universidad de La Frontera, Temuco, Chile. [2] Center for Research in Dental Sciences (CICO), Faculty of Dentistry, Universidad de La Frontera, Temuco, Chile.[3] Department of Pediatric Dentistry and Orthodontics; Faculty of Dentistry, Universidad de La Frontera, Manuel Montt #112, Temuco, Chile. [4]Center for Research in Epidemiology, Economics and Oral Public Health (CIEESPO), Faculty of Dentistry, Universidad de La Frontera, Temuco, Chile.[5] Laboratory of Molecular Microbiology and Antimicrobials, Department of Pathology and Experimental Therapeutics, School of Medicine, University of Barcelona, Barcelona, Spain. [4] Department of Pediatric Dentistry and Orthodontics; Faculty of Dentistry, Universidad de La Frontera, Manuel Montt #112, Temuco, Chile. [6] Department of Integral Adultos, Faculty of Dentistry, Universidad de La Frontera, Temuco 4780000, Chile; pablo.betancourt@ufrontera.cl.

*Correspondence:

[*]Eulàlia Sans-Serramitjana, Center of Plant, Soil Interaction and Natural Resources Biotechnology, Scientific and Biotechnological Bioresource Nucleus (BIOREN-UFRO), Universidad de La Frontera, Avenida Francisco Salazar 01145, PO Box 54-D, Temuco, 4780000, Chile. E-mail: eulalia.sans@ufrontera.cl. Telephone: (56) 45 232 5000.

[**]Pablo Betancourt, Center for Research in Dental Sciences (CICO), Faculty of Dentistry, Universidad de La Frontera, Temuco, Chile. E-mail: pablo.betancourt@ufrontera.cl. Telephone: (56) 45 273 4153.

Table S1. Search Strategies performed in electronic databases.

Database	Search Strategies
Medline	((Selenium AND nanoparticles) OR SeNPs OR NanoSe) AND (oral pathogens OR “Streptococcus mutans” OR Actynomyces OR “Candida albicans” OR “Porphyromonas gingivalis” OR Prevotella OR “Streptococcus oralis” OR Aggregatibacter OR “Enterococcus faecalis” OR “Fusobacterium nucleatum” OR bacteria OR oral biofilm OR oral infection)
Embase	((('selenium'/exp OR selenium) AND ('nanoparticles'/exp OR nanoparticles)) OR senps OR nanose) AND ((oral AND pathogens) OR 'streptococcus mutans' OR actynomyces OR 'candida albicans' OR 'porphyromonas gingivalis' OR prevotella OR 'streptococcus oralis' OR aggregatibacter OR 'enterococcus faecalis' OR 'fusobacterium nucleatum' OR bacteria OR (oral AND biofilm) OR (oral AND infection)) AND [embase]/lim
Scopus	(TITLE-ABS-KEY ("Selenium nanoparticles” OR senps OR nanose)) AND (TITLE-ABS-KEY (“oral pathogens” OR “Streptococcus mutans” OR actynomyces OR “Candida albicans” OR “Porphyromonas gingivalis” OR prevotella OR “Streptococcus oralis” OR aggregatibacter OR “Enterococcus faecalis” OR “Fusobacterium nucleatum” OR bacteria OR “oral biofilm” OR “oral infection”))
LILACS	(("Selenium nanoparticles" OR senps OR nanose) AND ("oral pathogens" OR "Streptococcus mutans" OR actynomyces OR "Candida albicans" OR "Porphyromonas gingivalis" OR prevotella OR "Streptococcus oralis" OR aggregatibacter OR "Enterococcus faecalis" OR "Fusobacterium nucleatum" OR bacteria OR "oral biofilm" OR "oral infection")

Table S2. Critical appraisal details.

Reference	Type of Study	I: Test substance identification	II: Test system characterization	III: Study design description	IV: Study results documentation	V: Plausibility of study design and data	Total points	It is considered reliable (yes/no)	Reliability category
[38]	In vitro	4	3	5	3	2	17	yes	1
[45]	In vitro	4	3	5	3	2	17	yes	1
[46]	In vitro	4	3	2	2	2	13	no	3
[47]	In vitro	4	3	6	2	2	17	yes	1
[48]	In vitro	4	3	6	3	2	18	yes	1
[55]	In vitro	4	3	6	3	2	18	yes	1
[49]	In vitro	4	3	4	1	2	14	no	3
[56]	In vitro	4	3	6	3	2	18	yes	1
[67]	In vitro	4	3	5	2	1	15	no	3
[43]	In vitro	2	2	3	2	0	9	no	3
[50]	In vitro	4	3	6	3	2	18	yes	1
[44]	In vitro	1	3	3	1	0	8	no	3
[68]	In vitro	4	3	6	3	2	18	yes	1
[57]	In vitro	4	3	6	3	2	18	yes	1
[51]	In vitro	4	2	5	2	2	15	yes	1
[58]	In vitro	3	2	5	3	2	15	yes	1
[59]	In vitro	4	2	5	3	2	16	yes	1
[52]	In vitro	4	2	6	2	2	16	yes	1
[53]	In vitro	4	2	5	2	2	15	no	3
[54]	In vitro	3	2	5	2	2	14	yes	2
[60]	In vitro	4	3	5	2	2	16	yes	1
[61]	In vitro	4	3	5	3	2	17	yes	1
[62]	In vitro	4	3	6	3	2	18	yes	1
[63]	In vitro	4	3	5	3	2	17	yes	1
[64]	In vitro	4	3	6	3	2	18	yes	1
[65]	In vitro	4	3	6	3	2	18	yes	1
[66]	In vitro	3	2	5	1	1	12	yes	2

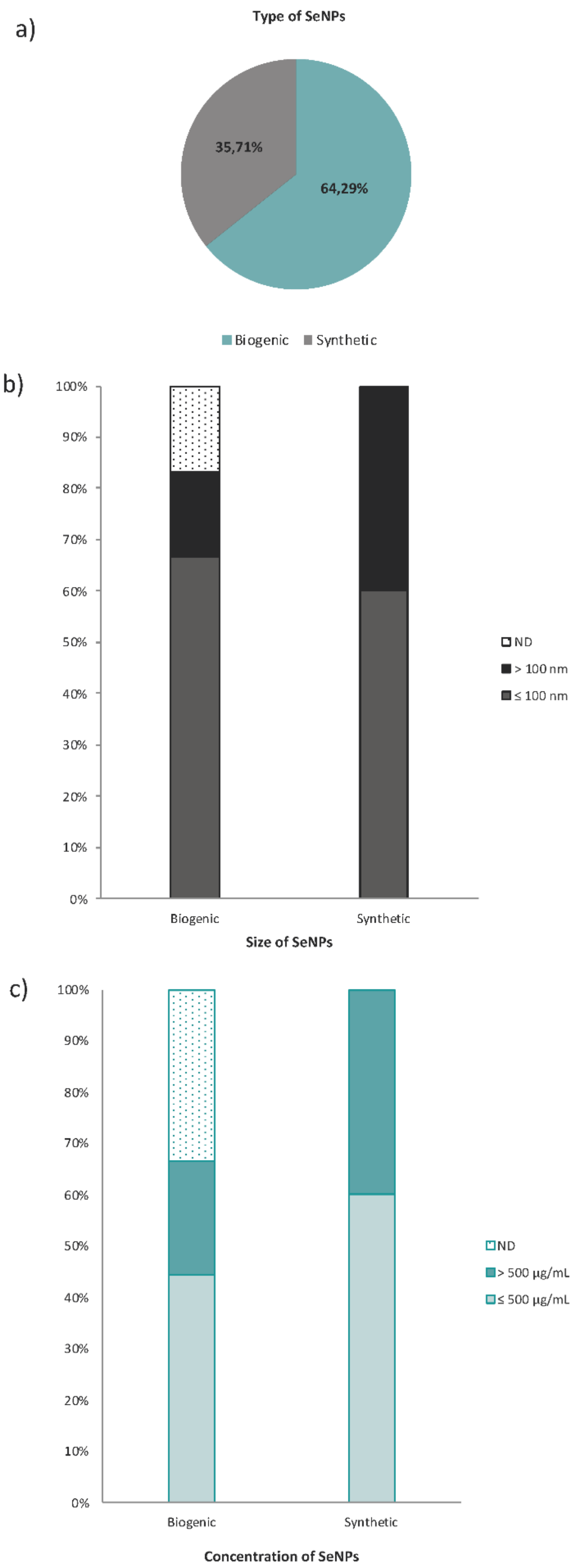

Figure S1. Type of SeNPs reported according to the synthesis methods employed (a), their size (b) and concentration tested (c). (ND, not determined).

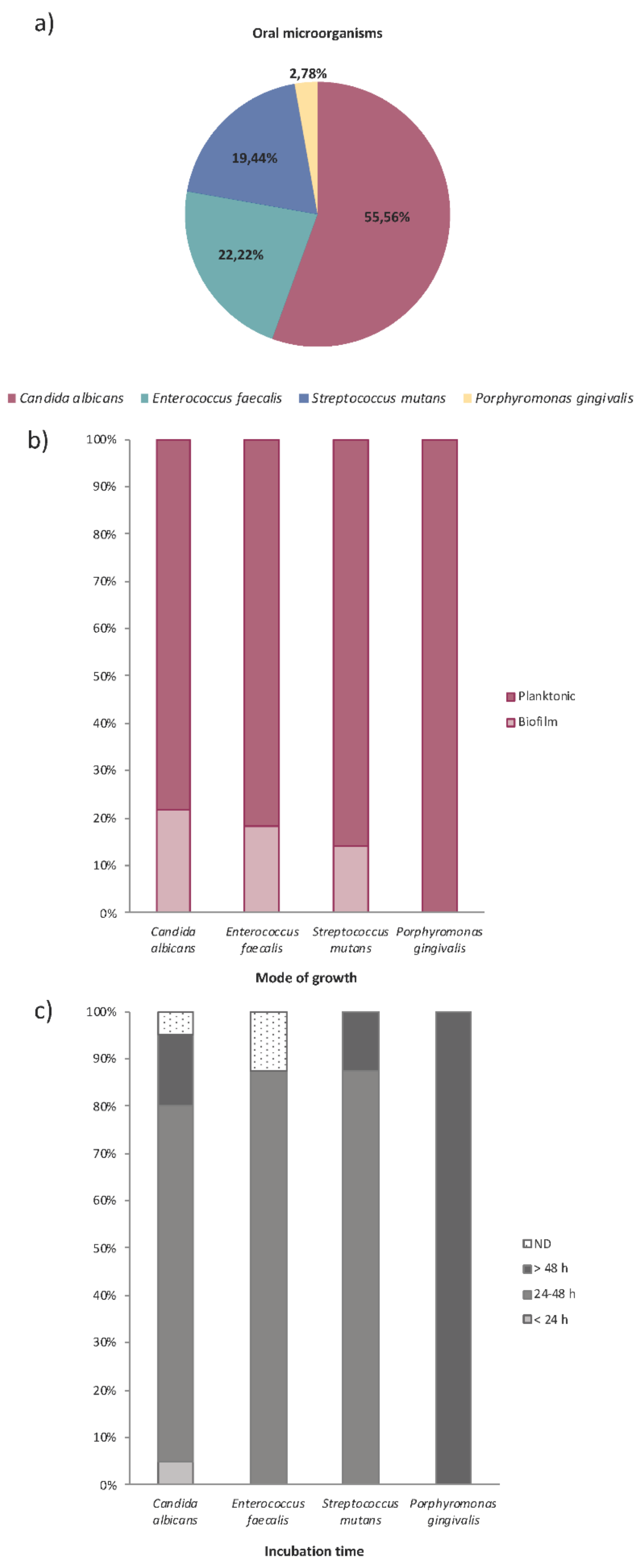

Figure S2. Oral microbial species investigated (a) according to their mode of growth (b) and the incubation time employed (c). (ND, not determined).
